# The Impact of *EPAC2*-Associated Junction Plakoglobin on Respiratory Syncytial Virus Infection

**DOI:** 10.3390/v17050627

**Published:** 2025-04-26

**Authors:** Chaitra A. Takle, Eun-Jin Choi, Eun Seok Choi, Devang Deepak, Kashish Khatkar, Jong Min Choi, Ke Zhang, Sung Yun Jung, Tian Wang, Wenzhe Wu, Xiaoyong Bao

**Affiliations:** 1 Department of Pediatrics, University of Texas Medical Branch, Galveston, TX 77555, USA; catakle@utmb.edu (C.A.T.); jinyjiny014@gmail.com (E.-J.C.); eunchoi@utmb.edu (E.S.C.); dedeepak@utmb.edu (D.D.); kakhatka@utmb.edu (K.K.); kezhang@utmb.edu (K.Z.); 2 Lester and Sue Smith Breast Center, Baylor College of Medicine, Houston, TX 77030, USA; jongmin.choi@bcm.edu; 3 Verna and Marrs McLean Department of Biochemistry and Molecular Pharmacology, Baylor College of Medicine, Houston, TX 77030, USA; syjung@bcm.edu; 4 Department of Microbiology and Immunology, University of Texas Medical Branch, Galveston, TX 77555, USA; ti1wang@utmb.edu; 5 Institute for Human Infections & Immunity, University of Texas Medical Branch, Galveston, TX 77555, USA; 6 Institute of Translational Science, University of Texas Medical Branch, Galveston, TX 77555, USA

**Keywords:** *EPAC2*, *JUP*, RSV, viral gene transcription and viral genome replication

## Abstract

Respiratory syncytial virus (RSV) is a leading cause of lower respiratory tract infections in infants, young children, and immunocompromised individuals. Currently, FDA-approved monoclonal antibody therapies are limited to infants and young children with severe RSV disease. As a result, there is an urgent need for comprehensive studies of RSV pathogenesis to support the development of new therapeutic strategies. Exchange proteins directly activated by cAMP (*EPAC*) have recently emerged as key regulators in various viral infections. Our previous work identified EPAC isoform 2 (*EPAC2*) as a critical factor in RSV replication and host innate immune responses. However, the molecular mechanisms underlying *EPAC2*’s role in RSV infection remain unclear. In this study, we investigated *EPAC2*-mediated RSV infection by identifying *EPAC2*-interacting proteins. Proteomics and immunoprecipitation analyses revealed that junction plakoglobin (*JUP*) interacts with *EPAC2* in both mock- and RSV-infected cells, with this interaction notably enhanced during RSV infection. To determine *JUP*’s role in RSV infection, we compared viral replication in *JUP*-deficient and control cells. *JUP* downregulation significantly reduced the production of infectious RSV particles, likely by impairing viral budding and viral gene transcription. Moreover, our findings indicate that *JUP* is essential for an effective cellular immune response to RSV infection. Together, these results suggest that *EPAC2* and *JUP* may cooperatively regulate RSV replication and dissemination.

## 1. Introduction

Respiratory syncytial virus (RSV) is a negative-strand RNA virus and the most common cause of lower respiratory tract infection (LRTI) in young children and infants [1]. Nearly 90% of infants are infected with RSV within the first two years of life, with the majority of hospitalizations occurring in otherwise healthy infants [1]. While vaccine development has shown promise, current efforts are primarily limited to protecting elderly individuals and pregnant women [2,3]. Nirsevimab remains the only available treatment, and its use is restricted to infants and select young children at increased risk for severe RSV disease [4]. Despite these advances, RSV continues to pose a significant global health challenge due to its considerable economic and clinical burden. This highlights the need for a comprehensive investigation into RSV disease mechanisms to identify therapeutic targets and develop effective strategies to reduce the severity of RSV infection.

Exchange proteins directly activated by cAMP (*EPAC*) are relatively novel guanine exchange factors (*GEFs*) that regulate a variety of biological processes [5]. *EPAC* proteins transduce signals primarily by activating small *GTPases*, predominantly *Rap1* and *Rap2,* which influence diverse downstream targets [5]. Initially recognized for their roles in cancer, neurological disorders, diabetes, and inflammation [6,7,8,9,10,11,12], *EPACs* have more recently emerged as key players in viral infections, with essential roles identified in at least seven human viruses [13,14,15,16,17,18]. Two main isoforms of *EPAC* exist in eukaryotic cells: *EPAC1* and *EPAC2*. *EPAC1* has been shown to favor replication of viruses such as Ebola, Middle East respiratory syndrome coronavirus (MERS-CoV), SARS-CoV-2, and influenza [14,15,18]. Our previous studies identified *EPAC2* as a promising therapeutic target for RSV infection; knockdown or knockout of *EPAC2*, as well as treatment with an *EPAC2*-specific inhibitor, significantly suppressed RSV replication and reduced the expression of pro-inflammatory cytokines and chemokines [13,16]. The critical role of *EPAC2* in RSV infection was further validated in mouse models [17]. Beyond RSV, *EPAC2* has also been implicated in pulmonary inflammation and tissue remodeling in response to cigarette smoke exposure, highlighting its importance in lung health [19]. However, the downstream targets of *EPAC2* in the pulmonary setting remain largely unknown. Identifying these targets could provide valuable insights into RSV pathogenesis and inform the development of novel therapeutic strategies.

Recently, we conducted a proteomics study to identify proteins that interact with *EPAC2* and discovered that junction plakoglobin (*JUP*) was among its associated partners. *JUP*, also known as *γ-catenin*, is a homolog to *β-catenin* and plays a key role in desmosome formation and cell-cell adhesion [20,21]. However, the functions of *JUP* in viral infection remain largely unexplored. Among the *EPAC2*-binding proteins identified, *JUP* exhibited the least enhancement in its interaction with *EPAC2* by RSV. We chose to focus on the *JUP*-*EPAC2* interaction based on the rationale that if this modest interaction could be experimentally validated, other *EPAC2*-associated proteins showing stronger binding might be even more biologically significant. *JUP* was also of particular interest due to its established functions. Silencing *JUP* has been shown to disrupt the structure of actin in the cell cytoskeleton^21^, and the cytoskeleton is known to play essential roles in multiple stages of viral infection, including the transport and assembly of viral proteins and particles, viral immune evasion, and cell-to-cell fusion [22,23,24,25]. Although *JUP*’s role in viral infection has not been defined, its influence on actin dynamics suggests it could impact RSV assembly and budding. Indeed, previous studies have shown that the disruption of actin inhibits RSV release [26], suggesting a potential link between *JUP* and viral egress. These findings suggest that *EPAC2* may regulate RSV assembly and budding, at least in part, through its interaction with *JUP*.

In this study, we demonstrated that silencing *JUP* through small-interfering RNA (siRNA) transfection in A549 cells led to a significant decrease in infectious particle production, viral gene transcription, and immune cytokine/chemokine secretion as compared to control samples transfected with non-targeting control siRNA (siCN). In addition, *JUP* controls replication-independent cellular inflammatory responses. Together, these results indicate that *JUP* may play a role in EPAC-2-mediated pathways that influence RSV infection.

## 2. Materials and Methods

### 2.1. Cell Lines and Virus Preparation

Human alveolar type II-like epithelial A549 cell line and human epithelial type II cell line Hep 2 were purchased from ATCC (Manassas, VA, USA). A549 cells, a common respiratory virus infection model, were cultured and maintained in F12K media with 10% FBS (vol/vol), 100 IU/mL penicillin, and 100 µg/mL streptomycin, as previously described [13,27]. These cell lines were maintained in incubators at 37 degrees Celsius and 5% CO_2_. Viral titer was determined by immunostaining in Hep-2 cells using polyclonal biotin-conjugated goat anti-RSV antibody (7950-0104; Bio-Rad, Hercules, CA, USA) and streptavidin peroxidase polymer (Sigma, St Louis, MO, USA) sequentially, as described [28,29].

### 2.2. EPAC2-Associated Proteins Preparation

A549 cells in 50% confluence were transfected with Flag-tagged *EPAC2* plasmid (a gift from Dr. Susumu Seino, Kobe University Graduate School of Medicine, Japan) using FuGENE 6^®^ Reagent (Promega, Madison, Wi, USA), according to the manufacturer’s protocol. Empty vectors were used as controls. After 30 h post-transfection, the cells were mock-infected or infected with RSV at an MOI of 1 for 15 h. The cells were then lysed by buffer 1 from the Immunoprecipitation Kit (# 11719386001, Roche, Indianapolis, IN, USA), followed by nonspecific cleaning using Protein G-agarose. The proteins in the lysis buffer 1 were then mixed with the monoclonal anti-Flag M2 antibody (F1804, Sigma, Saint Louis, MO, USA) or control and incubated at 4 °C for 4 h on a rocking platform, followed by Protein G resin overnight at 4 °C. The pellet beads by gravity sedimentation were then sequentially washed by Immunoprecipitation Washing buffers 2 and 3, according to the manufacturer’s protocol. An aliquot of the complex was then loaded into an SDS-PAGE gel to validate Flag-tagged *EPAC2* overexpression and immunoprecipitation.

The remaining protein complex was analyzed via mass spectrometry to Identify *EPAC2*-associated proteins. The beads were treated with sample loading buffer and subjected to SDS-PAGE using a 10% Bis-Tris gel. Protein bands were visualized using Coomassie Brilliant Blue staining, and the gel was segmented into four molecular weight fractions. Each gel fragment underwent in-gel digestion with 100 ng of trypsin in 20 µL of 50 mM NH_4_HCO_3_ at 37 °C overnight. Peptides were subsequently extracted with 100% acetonitrile, vacuum-dried, and reconstituted in 10 µL of 5% methanol containing 0.1% formic acid. The samples were then analyzed via nanoHPLC-MS/MS using an EASY-nLC1000 (Thermo Fisher Scientific, Waltham, MA, USA) coupled to an Orbitrap Fusion mass spectrometer (Thermo Scientific) with an electrospray ionization (ESI) source. A custom-built trap column packed with 3 µm Reprosil-Pur Basic C18 beads and a 5 cm × 150 µm capillary column packed with 1.9 µm Reprosil-Pur Basic C18 beads were employed for separation. The instrument, operated under Xcalibur software version 2.2 (Thermo Fisher Scientific), was set to data-dependent acquisition mode. MS/MS spectra were searched against a target-decoy Human RefSeq database (release 2015_06, containing 73,637 entries) using the Proteome Discoverer 1.4 interface (Thermo Fisher) with Mascot algorithm (Mascot 2.4, Matrix Science, Mount Prospect, IL, USA). The precursor ion mass tolerance was set to 20 ppm, with a fragment ion mass tolerance of 0.02 Da, allowing up to two missed cleavages. Peptide identifications were validated using Percolator with a false discovery rate (FDR) threshold of 1% based on q-values. Protein abundance was determined using iBAQ calculations derived from previously published methodologies [30]. Proteins that were not detected and lacked iBAQ values were imputed with half of the minimum detected value to facilitate *p*-value and fold-change calculations.

### 2.3. Western Blot

Western blot analysis was performed to validate EPAC-2-associated proteins, identified by the proteomics studies. In brief, Flag-tagged *EPAC2* that had undergone immunoprecipitation using an anti-Flag antibody was loaded into an SDS-PAGE gel. The anti-*JUP* antibody (A303-718A-T, Bethyl, Montgomery, TX, USA) was used to detect *JUP* in the pull-down complex. The membranes were stripped and reprobed by an anti-Flag antibody to validate the IP. The immunoprecipitation input was also checked by loading the total cell lysates to SDS-PAGE, followed by checking the expression of *EPAC2*-Flag, *JUP*, and β-actin.

The endogenous interaction between *EPAC2* and *JUP* was also examined. Briefly, A549 cells were either mock-infected or infected with RSV at a multiplicity of infection (MOI) of 1 for 15 h. Immunoprecipitation was performed as described above, using an anti-*EPAC2* antibody (Cat# 19103-1-AP, Proteintech, Rosemont, IL, USA) for pulldown, followed by detection of *JUP* in the *EPAC2* complex using an anti-*JUP* antibody (Cat# 13-8500, Thermo Fisher Scientific).

### 2.4. siRNA-Mediated Gene Silencing

A549 cells at 80-90% confluence were transfected with 100 nM siRNA specific for *JUP* (Table 1, Sigma) using Lipofectamine™ 2000 Transfection Reagent (Thermo Scientific) for 24 h or 48 h. Scrambled siRNAs were used as controls (Cat# SIC001, Sigma). The suppression efficiency of siRNAs was then confirmed by Western blot.

### 2.5. Cytokine/Chemokine Concentration Measurement

To quantify immune and inflammatory mediators in mock or RSV-infected samples, pellets were removed by centrifugation at 1000 rpm for 5 min. A 50 µL aliquot of the resulting supernatants was analyzed using the Bio-Plex multiplex system (Bio-Plex Pro Human Cytokine 27-plex Assay, Cat #M500KCAF0Y, Bio-Rad) to measure 27 kinds of cytokines and chemokines, including FGF-β, IL-2, IL-10, MIP-1α, Eotaxin, IL-4, IL-12, MIP-1β, G-CSF, IL-5, IL-13, PDGF-bb, GM-CSF, IL-6, IL-15, RANTES, IFN-γ, IL-7, IL-17A, TNF-α, IL-1β, IL-8, IP-10, VEGF, IL-1ra, IL-9, and MCP-1.

### 2.6. Virus Titration Assay

RSV harvested from A549 cell lysates or culture supernatants was diluted in a 5-fold serial dilution, followed by seeding 150 µL of it to Hep-2 cells, which were grown to confluence in 24-well plates (1.5 × 10^5^ cells/well). Plates were then put in a shaker for 1 h at 37 °C, 5% CO_2,_ and 50 rpm. After 1 h, 1 mL of MEM with 2% serum and 0.75% methylcellulose was added to each well, and the plates were kept in the incubator for 5 days (37 degrees Celsius, 5% CO_2_). After 5 days of incubation, the culturing gel solution was removed from the wells, and 0.5 mL of 10% formaldehyde was added to each well to fix the cells. After 30 min of incubation at room temperature, formaldehyde was removed from the wells, and 1 ml of 1% crystal violet (in EtOH) was added. After 30 min of incubation, the dye was removed, followed by plate washing and plaque counting to calculate the viral titers.

### 2.7. Quantitative Real-Time PCR (qRT-PCR)

To quantify RSV N gene expression, total cellular RNA was extracted by TRIzol reagents (Thermo Fisher Scientific). CDNA was synthesized with 1 μg of total RNA in a 20-μL reaction mixture using the TaqMan Reverse Transcription Reagents kit from ABI (catalog number N8080234; Applied Biosystems, Foster City, CA, USA). We used RT primer 5′-CTGCGATGAGTGGCAGGCTTTTTTTTTTTT*AACTCAAAGCTC*-3′. We incorporated a “tag” (underlined letters) as part of the assay due to self-priming exhibited by viral RNA. The tag sequence was derived from the bacterial chloramphenicol resistance (Cm^r^) gene. The sequence with bold letters is complementary to the poly(A) tails of the transcribed RSV N gene. The sequence in italics is N gene-specific. The reaction conditions were as follows: 25 °C for 10 min, 48 °C for 30 min, and 95 °C for 5 min. At a 25 °C annealing temperature, the 8 nucleotides (nt) matching N-specific sequences would not be sufficient for stable efficient priming of cDNA from the antigenome of hMPV. On the other hand, 20 nucleotides matching transcribed N (12 T’s and N gene-specific nucleotides) can attain stable annealing to the transcribed N gene. For Quantitative real-time PCR amplification, we used the RSV tag reverse primer CTGCGATGAGTGGCAGGC and the forward primer ACTACAGTGTATTAGACTTRACAGCAGAAG. The PCR was performed with 1 μL of cDNA in a total volume of 25 μL by using iTaq ^TM^ Universal SYBR Green Supermix (Cat# 1725124, Bio-rad, Hercules, CA, USA). The final concentration of the primers was 300 nM. 18S RNA was used as a housekeeping gene for normalization. PCR assays were run with the ABI Prism 7500 sequence detection system with the following conditions: 50 °C for 2 min, 95 °C for 10 min, and then 95 °C for 15 s and 60 °C for 1 min for 40 cycles. Duplicate cycle threshold (*C_T_*) values were analyzed in Microsoft Excel by the comparative *C_T_* (ΔΔ*C_T_*) method according to the manufacturer’s instructions (Applied Biosystems). The amount of target (2^−ΔΔ^*^CT^*) was obtained by normalization to the endogenous reference (18S) sample.

To quantify RSV antigenomic copies, synthetic transcripts of the genome were generated from the Topo plasmid containing N-P-M genes, using the T7 MegaScript kit, following the digestion with PmeI. The reaction mixture was then treated with Turbo Dnase and purified using the MegaScript kit. Primers were designed to span the N and P regions of the viral genome and incorporated a Cm^r^ tag. First-strand cDNA was transcribed with a P-specific primer, 5′-CTGCGATGAGTGGCAGGCACTACAGTGTATTAGACTTRACAGCAGAAG-3′. For PCR assays, we used RSV tag primer CTGCGATGAGTGGCAGGC and primer RSV P GCATCTTCTCCATGRAATTCAGG.

### 2.8. Reporter Gene Assays

To investigate the role of *JUP* in inflammatory responses, A549 cells in a 24-well plate were transfected in triplicate with 0.05 µg/well luciferase reporter gene plasmids containing multiple copies of NF-κB binding sites (NF-κB-Luc) and 100 nM siRNAs, either scrambled or *JUP*-specific, using lipofectamine 2000 according to the manufacturer’s protocol. At 24 h post-transfection, the cells were treated with TNF-α at a 20 ng/mL concentration per well. Following 24 h of treatment, the cells were lysed to measure luciferase reporter activity, using a SpectraMax iD3 microplate reader (Molecular Devices, San Jose, CA, USA). Cells without treatment were used as controls.

We also investigated the function of *JUP* in mediating RSV-induced cellular responses. In brief, A549 cells in 24-well plates were transfected with 0.5 µg/well luciferase reporter plasmids containing multiple copies of *IRF-3* binding sites of *IFN-β* (I*RF3*-Luc) and 100 nM siRNA, scrambled or *JUP*-specific, using lipofectamine 2000. A4 24 h post-transfection, the cells were mock-infected or infected with RSV at an MOI of 1 for 15 h, followed by cell lysis and luciferase activity measurement.

### 2.9. Statistical Analysis

The experimental results were analyzed using GraphPad Prism 5 software. An unpaired two-tailed *t*-test was employed to compare the difference. A *p*-value < 0.05 was considered to indicate a statistically significant difference. Single and two asterisks represent *p*-values of <0.05 and <0.01, respectively. Means ± standard errors (Ses) are shown.

## 3. Results

### 3.1. EPAC2-Associated Proteins

**Figure 1:**
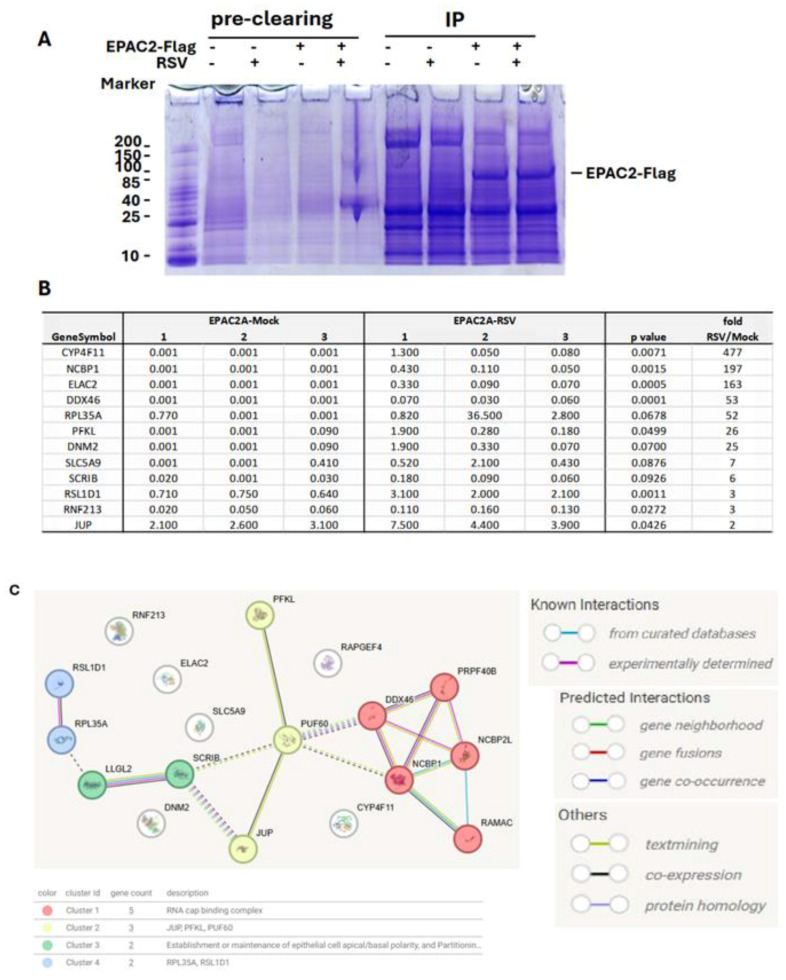
***EPAC2*-associated proteins.** Plasmid expressing Flag-tagged *EPAC2* was transfected into A549 cells, followed by mock or RSV infection. Cells with empty vectors were used as controls. Cells were lysed and subsequently precleaned with Protein G-agarose. The proteins in lysis were then mixed with the monoclonal anti-Flag M2 antibody, followed by Protein G resin, and washed. (**A**). An aliquot of the protein G-agarose complex was then loaded into an SDS-PAGE gel to validate the overexpression and immunoprecipitation. (**B**) The list of *EPAC2*-associated proteins and the impact of RSV infection on their interactions. (**C**) MSL clustering analysis using STRING. A defined number of clusters is based on the stochastic flow. The edges indicate both functional and physical protein associations. The line color indicates the type of interaction evidence. The dashed edges represent inter-cluster interactions.

The role of *EPAC* in viral infections is an emerging area of study. In infections caused by MERS-CoV, SARS-CoV-2, or influenza, *EPAC1* has been shown to promote viral replication [14,15,18]. Additionally, *EPAC1* facilitates Ebola virus uptake into vascular endothelial cells via micropinocytosis. Whether *EPAC2* contributes to viral infections remained unclear until our laboratory discovered its significant role in regulating both proinflammatory responses and RSV replication [13]. Most recently, in vivo experiments using RSV-infected mouse models further confirmed the critical involvement of *EPAC2* in prompting RSV replication and RSV-induced pulmonary inflammation [17]. We also recently reported a similar role of *EPAC2* in hMPV infection [16]. Despite these findings, the molecular mechanisms underlying *EPAC2*-mediated RSV infection remain largely unknown. To address this, we first employed a proteomics method to identify *EPAC2*-associated proteins. As shown in Figure 1A, *EPAC2*-Flag was successfully expressed and enriched using anti-Flag antibodies. Proteomic analysis identified eleven *EPAC2*-associated proteins; among them, eight exhibited significantly enhanced interaction with *EPAC2* following RSV infection, while three showed reduced interaction (Figure 1B). Notably, all listed proteins displayed no detectable binding in cells lacking *EPAC2*-flag expression. Further STRING network analysis revealed four natural clusters, identified using the MCL clustering method (which detects clusters based on stochastic flow) (Figure 1C).

### 3.2. JUP-EPAC2 Interaction

**Figure 2:**
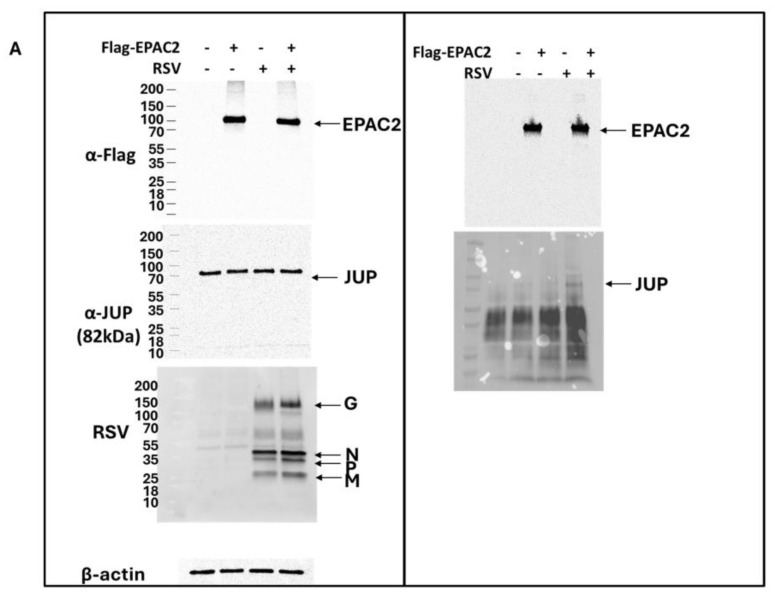

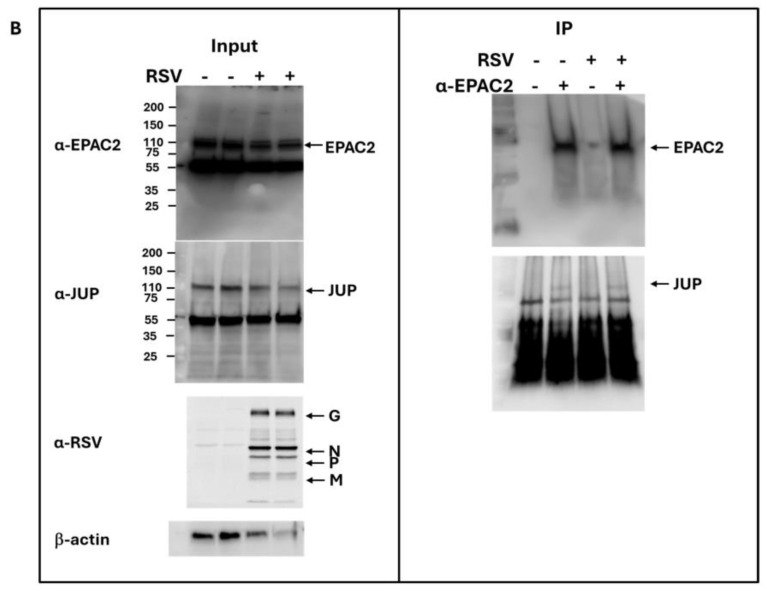
**Experimental confirmation of *JUP*-*EPAC2* interaction**. (**A**) The *EPAC2*-Flag overexpression and immunoprecipitation after mock or RSV infection were performed as described in Figure 1. (**Left**) panel: The input of overexpressed flag-tagged *EPAC2*, endogenous *JUP*, RSV proteins, and internal control β-actin were assessed by Western blot. (**Right**) panel: The presence of flag-tagged *EPAC2* and *JUP* in the immune precipitation complex following anti-Flag antibody and IgG resin treatment was detected by Western blot. (**B**). *EPAC2*-*JUP* interaction was also investigated using an anti-*EPAC2* antibody to pull down the endogenous *EPAC2*, followed by *JUP* detection in the IP complex. The IP experiments were repeated twice.

As shown in Figure 1B, *JUP* exhibited the least enhancement in its binding to *EPAC2* following RSV infection compared to other *EPAC2*-associated proteins. Based on this observation, we chose to begin our experimental validation by investigating the *JUP*-*EPAC2* interaction, reasoning that if this modest interaction could be confirmed, the stronger interactions observed for other proteins were likely to be biologically meaningful as well. An additional rationale for selecting *JUP* as our initial target was its known functional significance. Silencing *JUP* has been shown to disrupt actin organization within the cytoskeleton [21], and the cytoskeleton plays a critical role in multiple stages of viral infection, including the transport or assembly of viral proteins and particles, immune evasion, and cell-to-cell fusion [22,23,24,25]. As shown in Figure 2A. *JUP* was detected in the pull-down product using an anti-Flag antibody against Flag-tagged *EPAC2*, confirming the association of *EPAC2* and *JUP*. This interaction was observed even in the absence of RSV infection (bottom-right panel, second column) and was increased by RSV infection (bottom-right panel, last column).

To further validate this interaction under endogenous conditions, we performed immunoprecipitation using an anti-*EPAC2* antibody to pull down native *EPAC2* and assess its association with *JUP*. As shown in Figure 2B, the *EPAC2*-*JUP* interaction was confirmed in both mock- and RSV-infected cells.

### 3.3. The Roles of JUP in RSV Infection

**Figure 3:**
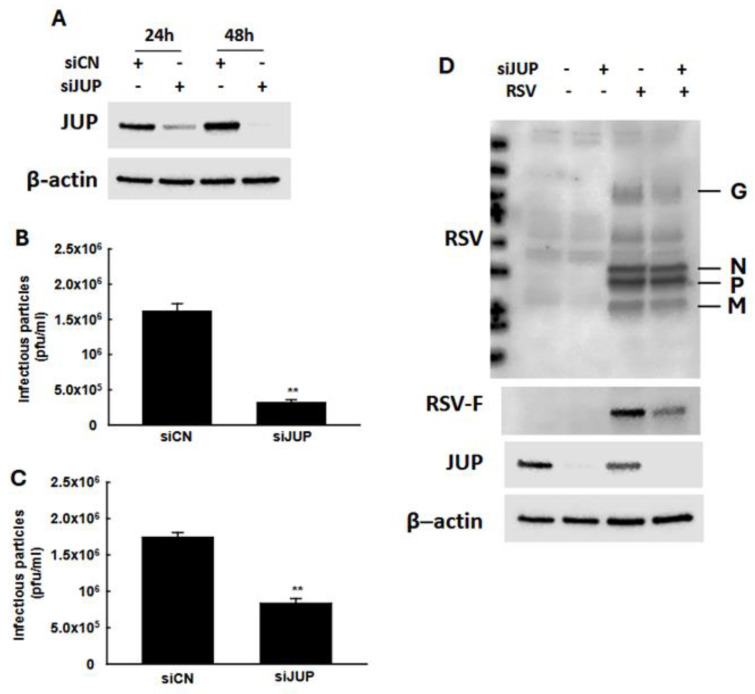
**The impact of *JUP* on RSV infection**. (**A**) After 24 h or 48 h post-transfection, siCN- or si*JUP*-transfected A549 cells were subjected to Western blot using anti-*JUP* antibody to check the efficiency of si*JUP* in downregulating *JUP* expression. (**B**–**D**), siCN- or si*JUP*-transfected cells were mock-infected or infected with RSV at an MOI of 1. After 2 h, the cells were washed with PBS twice and then incubated for 15 h. The immune staining using anti-RSV antibodies was used to quantify the infectious particles in the supernatant (**B**) and total lysed samples (**C**). Cell pellets were also prepared for Western blot to check the impact of *JUP* suppression on RSV protein expression. β-actin was used as a protein-loading control (**D**). The IP experiments were repeated twice. ** denotes *p* < 0.01, relative to siCN-treated and RSV-infected cells.

In our previous studies, we demonstrated that *EPAC2* facilitates both RSV replication and host inflammatory responses [13,16]. To investigate whether *EPAC2*-associated *JUP* contributes to RSV infection, we used *JUP*-specific siRNA to suppress its expression, followed by an assessment of the impact of *JUP* silencing on RSV replication. As shown in Figure 3A, treatment with *JUP*-specific siRNA at a concentration of 100 nM effectively reduced *JUP* expression, both at 24 h and 48 h post-transfection. We also found that the total number of infectious RSV particles produced by *JUP*-deficient cells was significantly lower than that of cells treated with scrambled control siRNA (siCN) (Figure 3B). Similarly, the number of infectious particles released into the supernatant was reduced in *JUP*-siRNA-treated cells compared to siCN-treated cells (Figure 3C). Notably, the reduction in infectious particles released into the supernatant (about 80%) was greater than the reduction observed in the total sample (about 50%), following *JUP* knockdown. Consistent with these findings, Western blot analysis using anti-RSV antibody confirmed that there was reduced viral protein expression in si*JUP*-treated cells compared to siCN-treated cells (Figure 3D).

**Figure 4:**
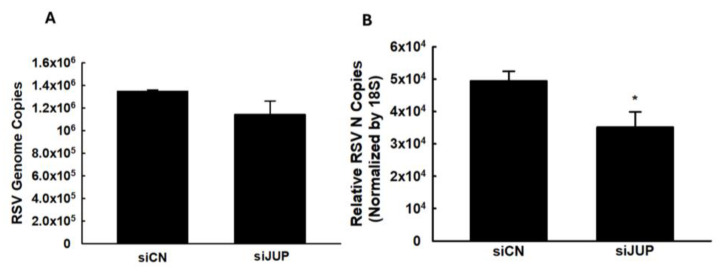
**The effect of *JUP* on RSV genome replication and gene transcription**. siCN- or si*JUP*-transfected A549 cells were harvested at 24 h p.i., followed by total RNA extraction. The viral genome copies (**A**) and RSV N gene transcription (**B**) were assessed by qRT-PCR. * denotes a *p* value of <0.5, relative to siCN-treated and RSV-infected cells. Four repeats were performed.

We also investigated whether *JUP* regulates RSV genome replication and viral gene expression. As shown in Figure 4, silencing *JUP* did not appear to affect viral genome replication; however, it significantly suppressed the expression of the N gene.

### 3.4. The Impact of JUP on RSV-Induced Cytokines and Chemokines

**Figure 5:**
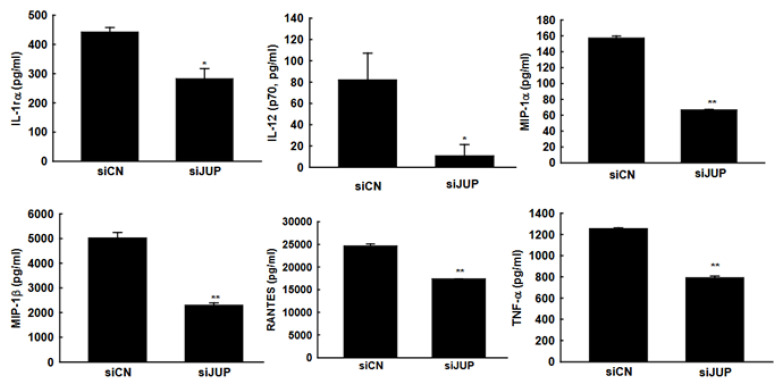
***JUP*-regulated cytokine and chemokine induction**. siCN- or si*JUP*-transfected A549 cells were mock infected or infected with RSV, as described in Figure 3. The cell pellets were obtained by centrifugation at 1000 RPM for 5 min, followed by supernatant harvesting. Bio-Plex multiplex assays quantified chemokines and cytokines in the supernatants. * and ** denote *p* values of <0.5 and <0.01, respectively, relative to siCN-treated and RSV-infected cells. 6 repeats were included.

In response to RSV infection, infected cells typically activate cellular responses by producing cytokines and chemokines. Our previous studies demonstrated that *EPAC2* promotes inflammation. To evaluate whether *JUP* suppression has a broader impact on RSV-induced secretion of proinflammatory and immunoregulatory molecules, we analyzed the secretion patterns of chemokines and cytokines in A549 cells with or without *JUP* silencing (Figure 5). Notably, *JUP* deficiency resulted in significantly lower levels of IL-1rα, IL-12 (p70), MIP-1α, MIP-1β, RANTES, and TNF-α at 15 h post-infection, compared to siCN-treated cells, supporting the importance of *JUP* in RSV-induced cellular responses. Unlike *EPAC2*, *JUP* did not affect the RSV-induced IP-10 and MCP-1 in A549 cells.

### 3.5. Modulation of Cellular Signaling by JUP

**Figure 6:**
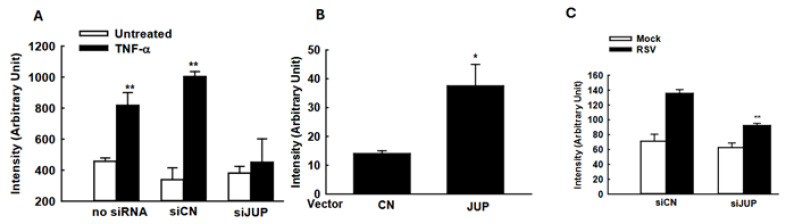
**The impact of *JUP* on cellular signaling**. (**A**) A549 cells in triplicate were transfected with 0.05 µg/well luciferase reporter gene plasmids containing multiple copies of *NF-κB* binding sites (*NF-κB*-Luc) and 100 nM siRNAs, either scrambled or *JUP*-specific, using lipofectamine 2000. At 24 h post-transfection, the cells were treated with *TNF-α* at a concentration of 20 ng/mL per well. Following 24 h of treatment, the cells were lysed to measure luciferase reporter activity. Cells without treatment were used as controls. ** denotes *p* value < 0.01, relative to paired mock-infected samples (white bars). (**B**) A549 cells were transfected with 0.5 µg/well luciferase reporter plasmids carrying multiple copies of the *IRF-3* binding site and 0.5 µg/well *JUP*-PCDNA3. At 15 h post-transfection, the cells were harvested for luciferase assay. * denotes a *p* value of <0.05, relative to paired cells with empty vector (CN). (**C**). A549 cells were transfected with 0.5 µg/well IFN-β luciferase reporter and 100 nM siRNAs specifically against *JUP* or scrambled siRNAs, by lipofectamine 2000. At 24 h post-transfection, the cells were mock-infected and infected with RSV for 15 hrs, followed by luciferase activity measurement. ** denotes a *p* value of <0.01, relative to siCN-treated RSV-infected cells; 3–4 repeats were performed.

The suppressed cellular responses could be an indirect outcome of the impaired RSV replication by *JUP* silencing. To investigate whether the *JUP* knockdown also leads to changes in inflammatory responses independent of viral replication, we used TNF-α to activate *NF-κB*, a transcription factor that plays an essential role in inducing inflammatory immune mediators in responses to viral infection, and investigated whether silencing *JUP* impacts NF-κB activation. As shown in Figure 6A, *JUP* knockdown significantly impaired *TNF-α*-induced *NF-κB* activation, in alignment with our previous finding on *EPAC2*-mediated NF-κB activation and current discovery on *EPAC2*-*JUP* interaction.

Many immune mediators also have *IRF-3* binding site(s) in addition to the *NF-κB* binding site. Therefore, we also investigated whether *JUP* affects cytokine/chemokine expression by regulating *IRF-3* activation. As shown in Figure 6B, *JUP* overexpression led to enhanced *IRF-3*-mediated luciferase expression. In addition, *JUP* knockdown significantly suppressed luciferase expression controlled by *IFN-β* transcription binding sites for *NF-κB* and *IRF-3* (Figure 6C).

## 4. Discussion

*EPAC*, a major cellular receptor for cAMP in addition to protein kinase A (*PKA*), plays major roles in cardiac diseases, cancer, neuronal differentiation, and respiratory inflammation [31,32,33,34]. In non-infectious disease models, *EPAC* has been reported to promote airway inflammation through the *EPAC*-activated mitogen-activated protein kinase kinase (*MAPKK*) pathway [35,36,37,38] or via *PLCε* [19]. Recently, the significance of *EPAC* in viral infections was also reported. In MERS-CoV, SARS-CoV-2, or influenza infection, *EPAC1* seems to promote viral replication [14,15,18]. Unlike these viruses, our laboratory discovered that it is *EPAC2*, but not *EPAC1*, which dominantly affects proinflammatory cellular/pulmonary responses to RSV, human metapneumovirus, and adenovirus [13,17], suggesting that the *EPAC* isoform evolved in viral infections is pathogen-dependent. However, the molecular mechanisms underlying *EPAC*-mediated viral infection are not well known, although such information is essential for developing novel therapeutic interventions against viral infections.

By comparing our previous study on *EPAC2*-mediated and the current study on *JUP*-mediated cellular responses to RSV infection, we found that *EPAC2* deficiency impacts a broader range of cytokines and chemokines than *JUP* deficiency, supporting the notion that *JUP* functions as one of the downstream signaling molecules of *EPAC2*. Unlike *EPAC2*, *JUP* did not affect the RSV-induced *IP-10* and *MCP-1* in A549 cells.

The biological functions of *JUP* were mostly investigated in the cardiac muscle. In cardiac muscle, *JUP* is a cytoplasmic component of desmosomes and adherens junctions and is, therefore, essential for its stability [39,40,41]. In cancer, *JUP* is involved in mediating the cell cytoskeleton actin [20,21]. The role of *JUP* in viral infection is not well known. We found that *JUP* deficiency led to a 50% reduction in total infectious particles and an 80% decrease in infectious particles in the supernatant, suggesting that *JUP* is critical for releasing infectious particles from cells. It has been previously reported that *actin* controls RSV budding [26]. The importance of actin in mediating virus budding out of cells is also reported for nonlytic rotavirus and SARS-CoV-2 [42,43]. Given the association between *JUP* and *actin*, it is not surprising to observe the impact of *JUP* on viral budding. Combined with our previous finding on viral entry independent of *EPAC2* [13], this finding may represent a mechanism by which *JUP* promotes viral replication via controlling virus budding.

The induction of many cytokines/chemokines is replication-dependent [44,45]. In addition to *JUP*-enhanced infectious particle production, we also discovered a slight but significant impact of *JUP* on N gene transcription, possibly leading to increased immune responses to RSV infection. However, we found that *JUP* could also control cellular responses in a replication-independent manner (Figure 6A, B), similar to our report demonstrating that *EPAC2* can control inflammation independent of RSV replication [13] and the report from others showing *EPAC2*-mediated non-viral respiratory diseases [19].

Based on *EAPC2*-associated proteins identified by immunoprecipitation/proteomics studies and Cluster analysis by STRING [46], we found that *JUP* potentially binds to RNA-binding proteins: the nuclear cap-binding protein subunit 1 (*NCBP1*) and *DDX46* (Figure 1C). *NCBP1* has been reported to play a crucial role in viral infections by interacting with viral mRNA and influencing antiviral responses [47,48]. *DDX46*, or *DEAD-Box Helicase 46,* is a protein that negatively regulates the innate antiviral response to viral infection [49]. Several proteins were not clustered into any groups, including *CYP4F11*, a cytochrome *P450* enzyme. It has been reported previously that the expression of *CYP4F11* can be influenced by virus-induced inflammation [50,51]. Some studies have shown that HCV-induced *CYP4F12,* another important member of *P450*, is bound to the HCV replication complex to facilitate viral replication [52]. In the future, we will continue to investigate whether these *EPAC2*-binding proteins are functional in regulating RSV infection.

In summary, this study revealed an important pathway that RSV may use to evade cellular responses, providing a potential therapeutic target for controlling RSV infection.

## Figures and Tables

**Table 1:** *JUP* siRNA Primers.

	5′-3′				
**Set 1**	Forward	CCAUUGUGCAUCUCAUCAA[dT][dT]			
	Reverse	UUGAUGAGAUGCACAAUGG[dT][dT]			
**Set 2**	Forward	GCAACCAUCGGCUUGAUCA[dT][dT]			
	Reverse	UGAUCAAGCCGAUGGUUGC[dT][dT]			

## Data Availability

All data generated/analyzed during this study are included in this published article. The datasets and related detailed methods are also available from the corresponding author upon request.
